# Oligodendrocyte Precursor Cell Transplantation into Organotypic Cerebellar Shiverer Slices: A Model to Study Myelination and Myelin Maintenance

**DOI:** 10.1371/journal.pone.0041237

**Published:** 2012-07-20

**Authors:** Jenea M. Bin, Soo Yuen Leong, Sarah-Jane Bull, Jack P. Antel, Timothy E. Kennedy

**Affiliations:** Department of Neurology and Neurosurgery, Montreal Neurological Institute, McGill University, Montreal, Quebec, Canada; Hannover Medical School, Germany

## Abstract

Current *in vitro* models to investigate the consequence of oligodendrocyte-specific loss-of-function mutations on myelination are primarily limited to co-culture experiments, which do not accurately recapitulate the complex *in vivo* environment. Here, we describe the development of an *in vitro* model of myelination and myelin maintenance in which oligodendrocyte precursor cells are transplanted into organotypic cerebellar slice cultures derived from dysmyelinated shiverer mice. Compared to neuron-oligodendrocyte co-cultures, organotypic slices more closely mimic the environment *in vivo*, while utilizing a genetic background that allows for straight-forward identification of myelin generated by transplanted cells. We show at the ultrastructural level that the myelin generated by wild-type transplanted oligodendrocytes is compact and terminates in cytoplasmic loops that form paranodal junctions with the axon. This myelination results in the appropriate sequestering of axonal proteins into specialized domains surrounding the nodes of Ranvier. We also demonstrate the applicability of this approach for xenograft transplantation of oligodendrocyte precursor cells derived from rat or human sources. This method provides a time-efficient and cost-effective adjunct to conditional knockout mouse lines or *in vivo* transplantation models to study oligodendrocyte-specific loss-of-function mutations. Furthermore, the approach can be readily used to assess the effect of pharmacological manipulations on myelin, providing a tool to better understand myelination and develop effective therapeutic strategies to treat myelin-related diseases.

## Introduction

Myelination of axons by oligodendrocytes is critical for achieving appropriate saltatory signal conduction in the central nervous system (CNS). Failure to properly myelinate during development, or remyelinate after injury, is characteristic of several myelin-related diseases including leukodystrophies and multiple sclerosis. Efforts to gain a better understanding of the complex process of myelination and to develop effective therapeutic treatments require the use of both *in vitro* and *in vivo* models. While techniques to study the development of oligodendrocyte precursor cells (OPCs) into mature oligodendrocytes have been well established *in vitro*, current models to study myelination *in vitro* pose several technical challenges and limitations that have restricted their utility.

Currently, two of the most commonly used *in vitro* methods to study CNS myelination are the organotypic slice culture and the neuron-glia co-culture systems (reviewed in [1]). Organotypic slice cultures, which can be grown either in a roller tube or on a semi-porous membrane, involve preparing slices of tissue from the postnatal brain [2]. Over a period of 2–4 weeks, endogenous OPCs within the slice mature and myelinate axons [3], [4]. In the co-culture method, isolated OPCs are seeded onto purified neuronal cultures, which the OPCs that successfully mature then proceed to myelinate. Several variations of myelinating co-cultures have been developed, which differ primarily in the type of neurons utilized [1]. Both of these systems have their advantages and limitations. For example, organotypic slice cultures maintain a microenvironment that more closely resembles the *in vivo* environment and achieve more robust myelination than the co-culture system. On the other hand, the co-culture system provides the flexibility to use oligodendrocytes and neurons from different genetic backgrounds, which can be useful to study cell-specific defects arising from loss- or gain-of-function mutations. Here, we describe the development of a new protocol that combines these advantages from both systems to study oligodendrocyte-specific defects in myelination and myelin maintenance *in vitro*.

Transplantation of OPCs into the shiverer mouse brain is a well established technique employed to study cell replacement and myelination *in vivo* (reviewed in [5]). The shiverer mouse genome contains a large deletion in the myelin basic protein (MBP) gene that results in the failure to produce all six classic protein isoforms of MBP, and consequently causes extensive CNS dysmyelination [6]. Thus, any myelin produced by wild-type OPCs transplanted into a shiverer CNS can be unambiguously identified by positive immunohistochemical staining for MBP. Using a similar principle *in vitro*, we show that allogenic OPCs transplanted into homozygous shiverer organotypic cerebellar slice cultures achieve robust myelination, and further show that this myelin results in the proper sequestering of axonal proteins into specialized domains that surround nodes of Ranvier. We have maintained such slices in culture for more than 64 days post-transplant, making this model useful for studying both myelination and myelin maintenance.

## Materials and Methods

### Ethics Statement

All procedures with animals were approved by the Montreal Neurological Institute Animal Care Committee (approval ID #4330) and performed in accordance with the Canadian Council on Animal Care guidelines for the use of animals in research. Studies using human fetal CNS tissue or human adult CNS tissue were approved by the Albert Einstein College of Medicine Institutional Review Board (approval ID #1993-042) and the Montreal Neurological Institute and Hospital Research Ethics Board (approval ID # ANTJ 1988/3). Informed written consent was received from all tissue donors.

### Animals

Shiverer mice [6] were obtained from Dr. Alan Peterson (McGill University) and bred into a C57/Bl6 background. For the isolation of wild-type OPCs, newborn CD1 mouse pups and Sprague-Dawley rat pups were obtained from Charles River Canada (Montreal, Canada).

### Preparation and culture of organotypic slices from shiverer mice

Organotypic cerebellar slice cultures were prepared from P0 shiverer pups as previously described [3], [4]. Briefly 200 µm sagittal slices of cerebellum were sectioned using a McIlwain tissue chopper and transferred onto Millicell cell culture inserts (Millipore, MA, USA) in a 6-well plate containing 1 mL of serum-containing medium (SCM; 50% MEM with Earle's Salts, 25% heat inactivated horse serum, and 25% Earle's balanced salt solution supplemented with glutamax, penicillin-streptomycin, 6.5 mg/mL glucose, and fungizone). The media was changed every two days during the culture period. For all experiments, except where specified, beginning with the second media change the cultures were gradually switched to serum-free medium (SFM; 50% DMEM and 50% F12 supplemented with 1% B27, 0.5% N2, glutamax, penicillin-streptomycin, and fungizone) [7]. This was done by mixing 1/3 SFM with 2/3 SCM on day 4 *in vitro* and 2/3 SFM with 1/3 SCM of day 6 *in vitro*. From 8 days *in vitro* (DIV) onward, SFM was used.

### Preparation of mouse glial cultures

Mixed glial cultures were prepared from P0 CD1 wild-type mouse pups as described [8]. In brief, cortices were dissected in ice cold MEM/HEPES and digested with papain (1.2 U/mL), L-cysteine (0.24 mg/mL) and DNAseI type IV (40 µg/mL). Dissociated cells were plated in PDL-coated T25 flasks (cortices from one pup/flask) with DMEM media supplemented with 10% FBS, penicillin-streptomycin, and glutamax. Media was changed on the fourth day, and every three days thereafter. Beginning with the second media change, 5 µg/mL of insulin was also added to the media to enhance the number of OPCs.

### Preparation of rat glial cultures

Mixed glial cultures were prepared from P0 Sprague Dawley rat pups as described [9]. In brief, cortices were dissected in ice cold HBSS and digested with tryspin-EDTA and DNAseI. Dissociated cells were plated in 10 PDL-coated T75 flasks with DMEM media supplemented with 10% FBS, penicillin-streptomycin, and glutamax. Media was changed every 2–3 days.

### Isolation of mouse and rat oligodendrocyte precursor cells

OPCs were collected by shake-off and differential adhesion after 8–14 DIV as described [10]. Cultures were agitated on an orbital shaker for 1 hr at 150 rpm, 37°C to remove loosely adherent microglia. All of the media was replaced and flasks were then allowed to equilibrate in the tissue culture incubator for 1 hr. Flasks were then shaken for 14–16 hrs at 180 rpm to detach the OPCs. Detached cells were plated in uncoated petri dishes for 30 min, during which time the majority of contaminating microglia attached to the surface of the plate, while the OPCs remained floating in the medium. In some experiments, 10 µM of CMFDA cell-tracker dye (Invitrogen, ON, Canada) was added to the media during this incubation period to label the cells. Floating cells were then collected, pelleted and resuspended to a density of 20,000 cells/µL in oligodendrocyte defined medium (OLDEM; DMEM, 5 µg/mL insulin, 100 µg/mL transferrin, 30 nM sodium selenite, 30 nM triiodothyronine, 100 µg/mL penicillin-streptomycin, 2 mM glutamax) in preparation for transplantation. Fluorescent activated cell sorting identified >95% of isolated mouse OPCs to be PDGFαR+.

### Isolation of human fetal and adult oligodendrocyte precursor cells

Human fetal CNS tissue was obtained from 15- to 16-week-old embryos provided by the Human Fetal Tissue Repository (Albert Einstein College of Medicine, NY, USA). Human adult CNS tissue was collected via surgical resection performed as treatment for non-tumour-related intractable epilepsy in accordance with guidelines from Biomedical Ethics Unit of McGill University. Tissues were dissociated with 0.25% trypsin and 25 µg/mL DNase I at 37°C for 30 min and then passed through a nylon mesh. Adult CNS cell suspension was subjected to an additional cell separation step using a linear 30% Percoll gradient (Pharmacia Biotech, NJ, USA) to remove myelin debris and red blood cells. Fetal cells were collected from pre-myelinating brain and hence did not required Percoll gradient separation. Total neural cells were cultured overnight (fetal cells) or for 48 hrs (adult cells) in DMEM/F12 media supplemented with N1 and BSA. Floating cells were harvested and labelled with PE-conjugated mouse anti-O4 (FAB1326P, R&D systems, MN, USA) and collected by fluorescence activated cell sorting.

### Transplantation of OPCs into shiverer slices

Transplantations were performed into shiverer^−/−^ slices that had been cultured 10–21 DIV. 20,000 cells were injected into each slice in two 0.5 µl injections using a broken glass-pulled pipette tip. Slices were cultured for 3–9 weeks before fixing for further analysis.

### Immunohistochemistry

Slices were fixed in 4% paraformaldehyde (PFA) in PBS at pH 7.4 for 1 hr on ice (or for 15 min at room temperature for Na^+^ channel or Kv1.2 immunostaining). Slices were blocked in 3% heat-inactivated horse serum, 2% bovine serum albumin (BSA), 0.25% triton X-100 for a minimum of 2 hrs. Primary antibody was prepared in 2% BSA, 0.25% triton X-100 PBS and incubated on the slices for 36–48 hrs, after which they were washed 1×10 min, 2×1 hr in PBS. Secondary antibody was prepared in 2% BSA PBS and incubated on the slices for 16–24 hrs. Slices were washed 1×10 min, 2×1 hr, 1 x overnight in PBS before mounting with Fluoromount G. Primary antibodies used included: rat anti-MBP (1∶100; Millipore, MA, USA), chicken anti-MBP (1∶1000; Aves Labs, OR, USA), mouse anti-caspr (1∶50; NeuroMab, CA, USA), rabbit anti-caspr (1∶1000; gift from Dr. David Coleman, Quebec, Canada), mouse anti-Na^+^ channel (1∶300; Sigma, MO, USA), rabbit anti-Kv1.2 (1∶300; Alomone Labs, Jerusalem, Israel), chicken anti-NFM (1∶1000; Aves Labs, OR, USA), and mouse anti-axonal neurofilaments (SMI-312; 1∶500; Covance, NJ, USA). Secondary antibodies used included: Alexa 488 or 555 donkey anti-rabbit, alexa 488 donkey anti-rat, alexa 546 goat anti-chicken, alexa 488, 555, or 647 donkey anti-mouse (1∶1000, Molecular Probes, OR, USA).

### Electron Microscopy

Slices were fixed in 2.5% gluteraldehyde in 0.1 M sodium cacodylate buffer for a minimum of 24 hrs to a maximum of 2 weeks. Samples were then osmicated with potassium ferrocyanide-reduced 1% osmium tetraoxide solution for 1 hr, dehydrated with successive rinses of increasing concentrations of ethanol, then infiltrated and embedded in Epon. 70–100 nm sections were mounted onto 200 mesh copper grids and stained with 4% uranyl acetate for 5 min followed by Reynold's lead citrate for 3 min. The sections were examined using a FEI Technai 12 transmission electron microscope at 120 kV and images collected using a Gatan Ultrascan 4k×4k digital (CCD) camera system.

## Results

### Optimization of the transplant protocol

In order to generate an *in vitro* model of myelination with the capacity to use neurons and oligodendrocytes from different genetic backgrounds, while maintaining an environment that closely resembles that *in vivo*, we transplanted isolated mouse OPCs into organotypic cerebellar slices generated from shiverer mutant mice. Homozygous shiverer mutant mice do not express MBP. As a result, oligodendrocytes mature and sometimes ensheath axons, but are unable to form compact myelin [6]. This provides an ideal background to quickly and unambiguously identify myelin generated by transplanted cells using immunohistochemical staining for MBP. In addition, any compact myelin with major dense lines detected by electron microscopy can be concluded to have been generated by transplanted oligodendrocytes.

In initial studies, we transplanted wild-type OPCs into organotypic cerebellar slices prepared from P0 shiverer mice that were cultured for 2–5 DIV in standard organotypic slice culture media, which typically contains 25% serum. However, at 21 days post-transplant, we found few, if any, MBP-positive myelinating oligodendrocytes in the cultures, suggesting that the OPCs had either failed to be incorporated into the slice, failed to survive, or failed to mature into oligodendrocytes. Using a fluorescent cell-tracker dye to follow the fate of the transplanted cells, two factors were found to play a critical role in the efficiency of myelination: (1) the age of the slice at transplantation and (2) the composition of the media.

**Figure 1:**
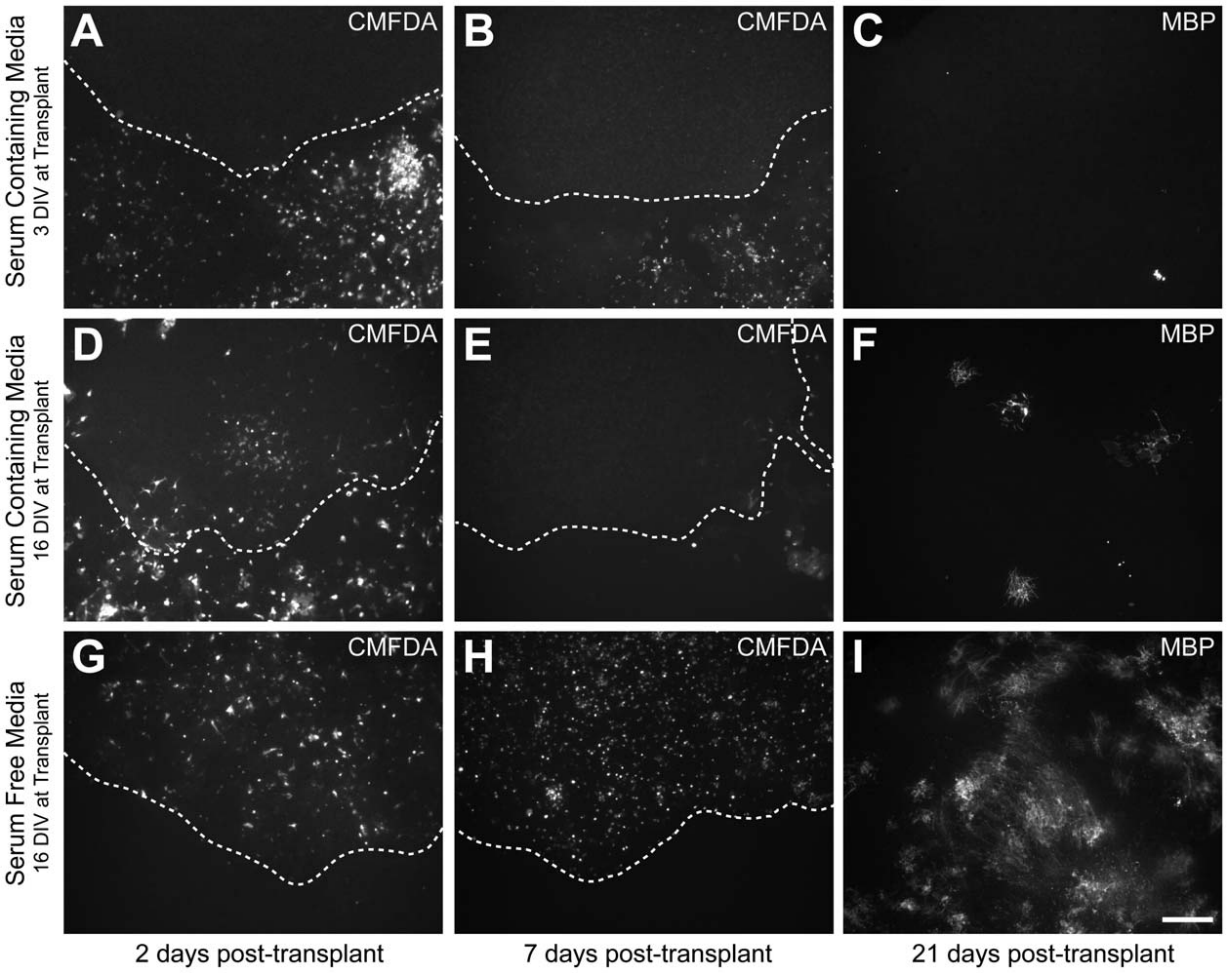
Increased myelination efficiency by transplanted OPCs in older slices and serum-free media. Representative images comparing transplantation of CMFDA-labelled OPCs into 3 DIV organotypic slices cultured in serum-containing media (A–C), 16 DIV organotypic cerebellar slices cultured in serum-containing media (D–F), and 16 DIV organotypic cerebellar slices cultured in serum-free media (G–I). In both of the serum-containing media conditions, substantial migration of the labelled OPCs out of the slices onto the surrounding membrane could be observed by two days post-transplant (A,D). More cells remained in the slices transplanted at 16 DIV (D), compared to the slices transplanted at 3 DIV (A); however, by 7 days post-transplant few of these labelled cells remained (B,E). In contrast, when transplanting into slices cultured in serum-free media, the majority of cells remained within the slices at both 2 and 7 days post-transplant (G,H). After 3 weeks, substantially more MBP-positive myelin was present in the serum-free media condition (I), compared to both serum-containing media conditions (C,F). The dashed line shows the boundary between the slice (above) and the culture membrane (below). Scale bar = 200 µm.

Unsuccessful incorporation of OPCs into the slice was identified to be the principle cause for the failure of these cells to myelinate. While at twelve hours post-transplant, labelled OPCs were dispersed throughout the slice, when examined two days post-transplant, virtually all labelled cells had migrated out of the slice onto the surrounding membrane (Figure 1A–C). Transplanting into slices that had been cultured for a longer period of time *in vitro* increased the number of cells that remained within the slice, as well as the amount of myelination; however, the majority of cells still migrated out of the slices (Figure 1D–F).

**Figure 2:**
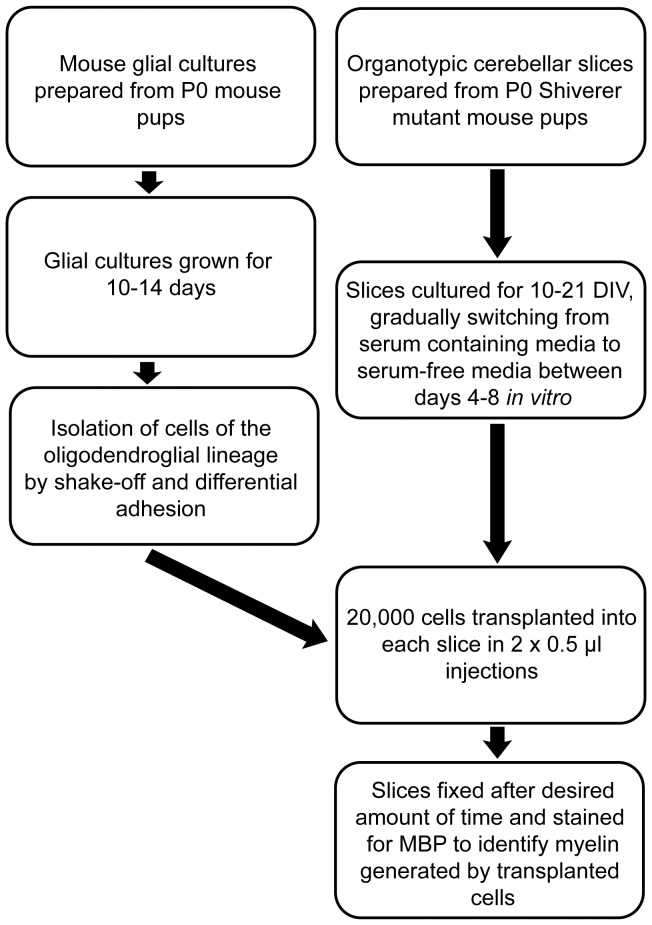
Overview of the OPC – organotypic cerebellar slice culture transplant protocol.

Replacing the standard serum-containing media (SCM) with a serum-free defined media (SFM) (see Material and Methods for detailed description of media) was crucial to prevent OPC migration out of the slice, and resulted in a robust increase in the amount of myelination by the transplanted cells. At two days post-transplant, while the majority of OPCs had migrated out of the slice in the SCM condition (Figure 1D), in the SFM condition, transplanted OPCs remained distributed throughout the slice (Figure 1G). By seven days post-transplant, a large number of transplanted cells were still readily observed in the SFM condition, whereas in the SCM condition few labelled cells remained (Figure 1E,H). Immunostaining fixed slices for MBP-positive myelin three weeks post-transplant revealed a greater amount of myelin generated by the transplanted cells in the SFM condition compared to the SCM condition (Figure 1F,G). We concluded that SFM was more conducive to OPC incorporation into the slice, and consequently, all subsequent experiments were conducted using slices cultured in SFM, as outlined in Figure 2.

### Robust production of compact myelin by transplanted OPCs

**Figure 3:**
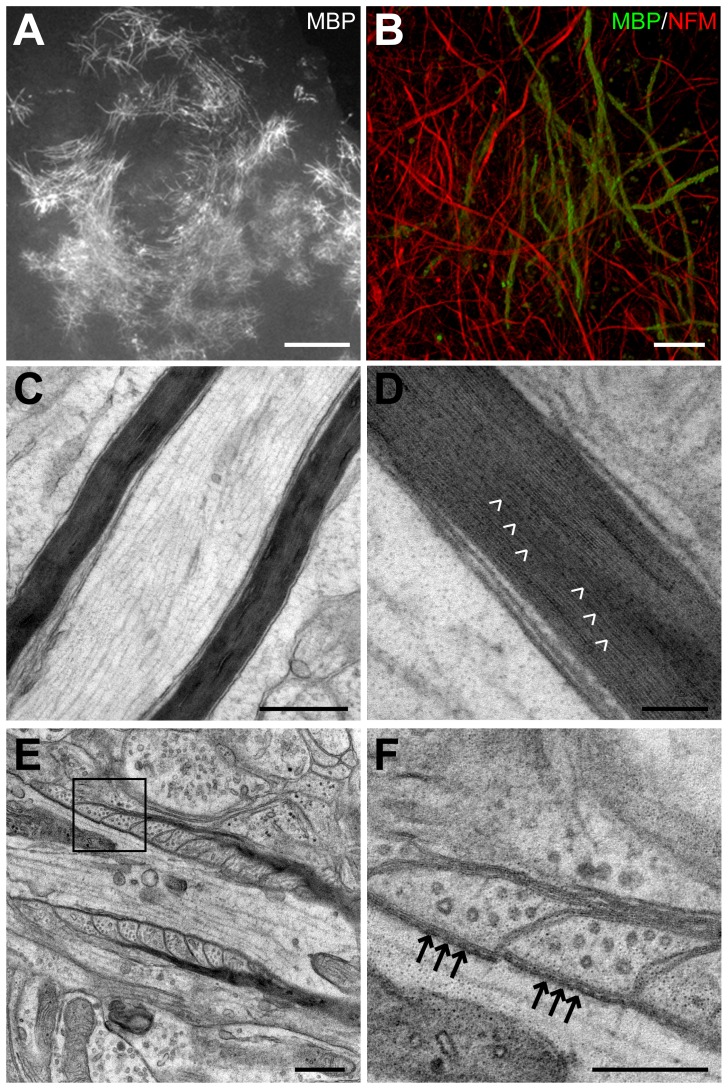
Myelin formed by the transplanted cells is compact and has appropriate paranode ultrastructure. (A) Low magnification epifluorescent and (B) high magnification confocal images showing that at three weeks post-transplant, the majority of MBP-positive oligodendrocytes exhibited the morphology of mature myelinating oligodendrocytes based on the presence of tubular shaped MBP-positive segments that surrounded NFM-labeled axons. (C,D) Electron microscopy of shiverer mutant organotypic slices transplanted with wild-type mouse OPCs showing that myelin formed by the transplanted OPCs is compact. This myelin contains major dense lines (D; white arrowheads), which differentiates it from membrane ensheathments made by shiverer oligodendrocytes. (E) Paranodes of myelin formed by transplanted cells appear ultrastructurally normal by electron microscopy. (F) Magnification of boxed area in E, displaying that transverse bands (black arrows) are present and regularly spaced. Scale bars: A = 100 µm; B = 20 µm; C = 500 nm; D = 100 nm; E = 500 nm; F = 250 nm.

Initial ensheathment of axons by MBP-positive myelin membrane could be observed in transplanted slices at two days post-transplant. By two to three weeks post-transplant, the majority of MBP-positive cells within slices exhibited the morphology of mature myelinating oligodendrocytes, based on the presence of multiple tubular shaped MBP-positive segments that surrounded axons and on the absence of superfluous non-myelinating MBP-positive processes (Figure 3A,B). The presence of compact myelin in the transplanted slices was confirmed by electron microscopy at both five and nine weeks post-transplant (Figure 3C,D). In agreement with previous studies, no compact myelin was found in the untransplanted shiverer organotypic slices [6], . Paranodes also formed properly, with compact myelin terminating in cytoplasmic loops in close contact with both the axon and with neighbouring glial loops (Figure 3E). Transverse bands, which are specialized contacts that link paranodal loops to the axon, were also readily detected (Figure 3F).

### Formation of specialized domains along axons myelinated by transplanted oligodendrocytes

**Figure 4:**
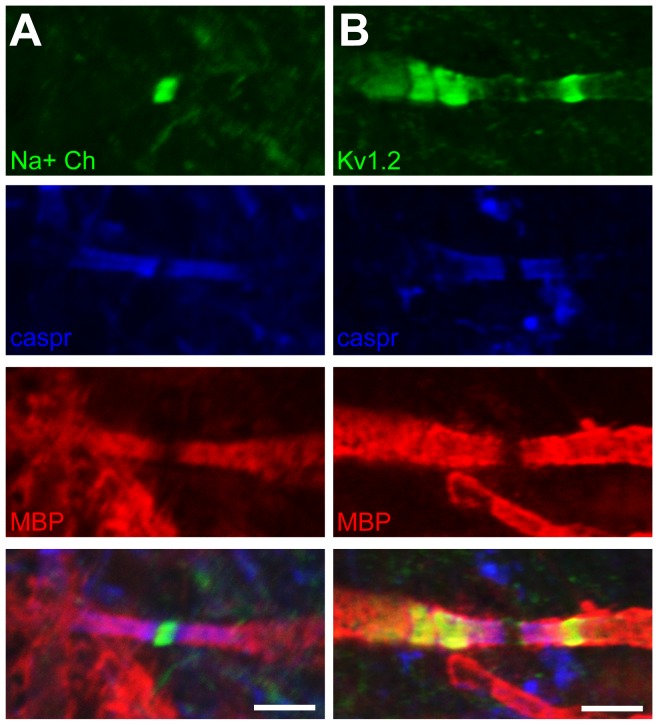
Axons myelinated by transplanted OPCs display appropriate specialized domain structure around nodes of Ranvier. (A) Sodium channels sequestered at the node. (B) Kv1.2 channels sequestered at the juxtaparanode. (A,B) Caspr sequestered at the paranode. Flanking MBP staining indicates myelin is generated by transplanted OPCs. Scale bar = 3 µm.

Myelination results in the formation of specialized domains along the axon: nodes of Ranvier, paranodes, juxtaparanodes, and internodes [12]. Using immunohistochemical staining and confocal microscopy, we determined that myelin formed by the transplanted oligodendrocytes resulted in the appropriate sequestering of sodium channels at the node of Ranvier, caspr at the paranode, and Kv1.2 channels at the juxtaparanode (Figure 4A,B). Sodium channel sequestering was readily observed by four days post-transplant, while caspr channel sequestering appeared complete by approximately ten days post-transplant. Maintenance of these molecular domains was observed for at least 64 days post-transplant. This axonal domain specialization, taken together with the correct paranodal ultrastucture, makes this a useful model for examining the specific contribution of oligodendrocytes to the formation and maintenance of paranodes and the neighbouring domains found along axons.

### Xenotransplantation of OPCs derived from rat and human brain

**Figure 5:**
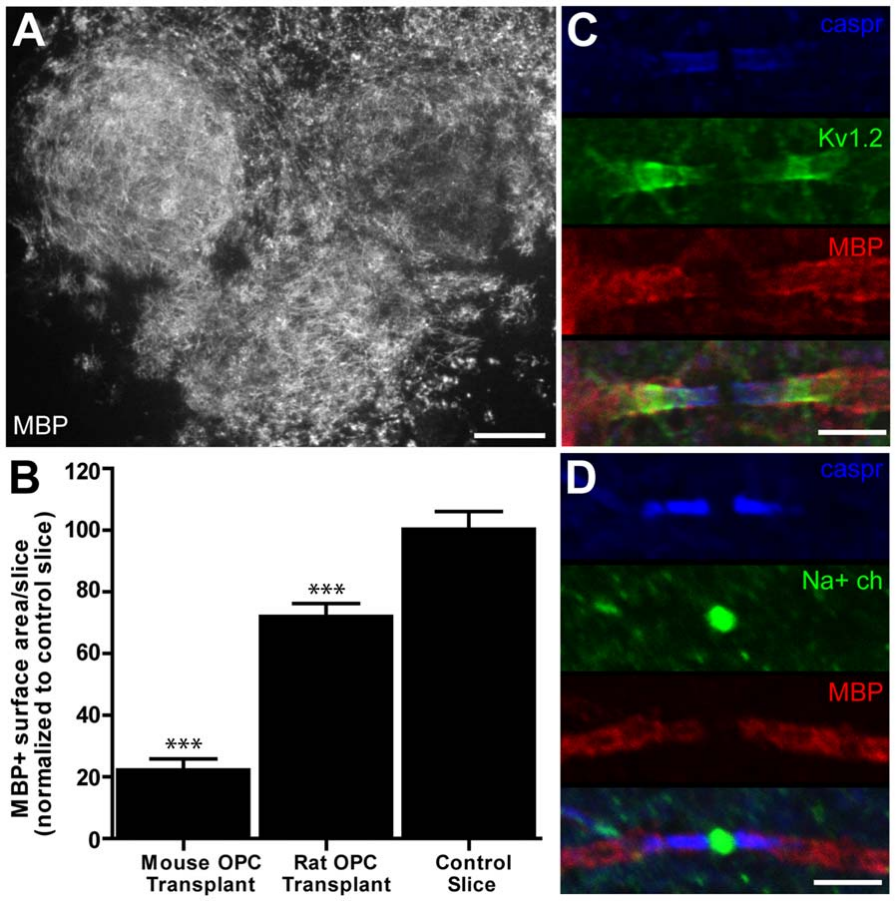
Rat OPCs myelinate shiverer organotypic cerebellar slices with high efficiency. (A) Extensive myelination throughout a shiverer organotypic slice by transplanted rat OPCs. (B) Quantification of MBP-positive surface area in shiverer organotypic slices transplanted with wild-type mouse OPCs and with wild-type rat OPCs, compared to myelinated control organotypic slices. (C) Sequestering of Kv1.2 at juxtaparanode and caspr at paranode of axons myelinated by rat OPCs. (D) Sequestering of sodium channels at the node and caspr at the paranode of axons myelinated by rat OPCs. Scale bars: A = 400 µm; C & D = 3 µm. *** p<0.0001 one-way ANOVA with Tukey's post-hoc test.

In some experimental paradigms it may be of interest to transplant OPCs generated from species other than mouse. For example, when the use of transgenic mice is not necessary, it may be advantageous to use wild-type rat OPCs, which, when compared with mouse OPCs, can be obtained in higher numbers per litter (approximately 5x more in our experience) and cultured more readily *in vitro*. Transplantation of OPCs obtained from newborn rat pups into shiverer organotypic cerebellar slices resulted in robust myelination and appropriate sequestering of proteins surrounding the nodes of Ranvier (Figure 5A,C,D). In all experimental trials, rat OPCs generated more MBP-positive myelin (71% compared to control) than mouse OPCs (22% compared to control), with some transplanted slices displaying amounts of myelination that resembled what was typical for untransplanted wild-type control slices (Figure 5B). Thus, our findings indicate that rat OPCs can be substituted for mouse OPCs in this protocol.

**Figure 6:**
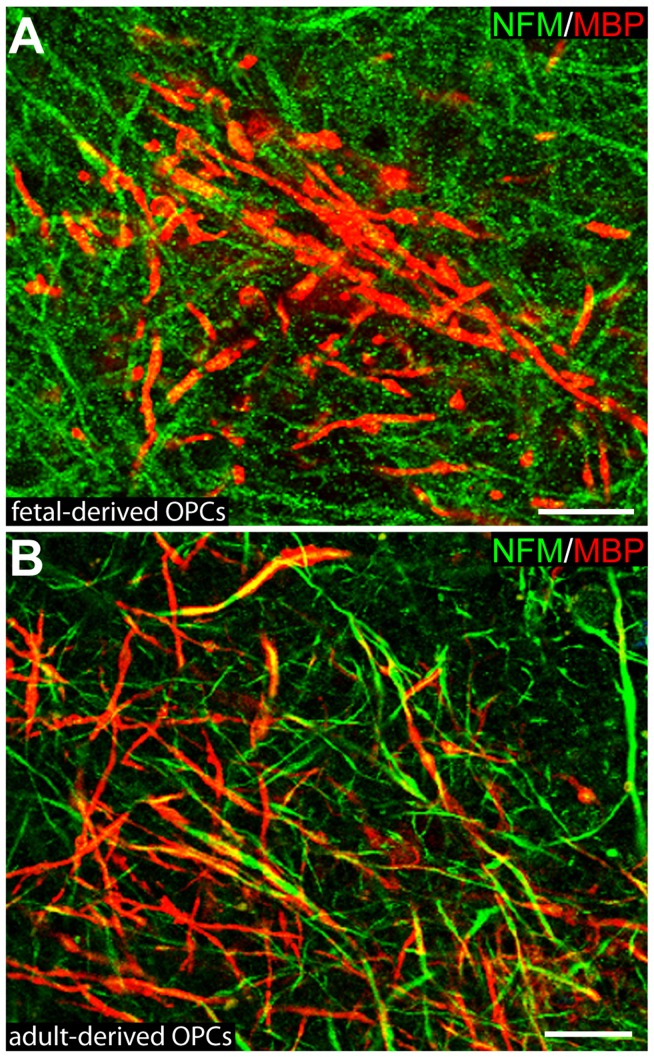
Human-derived OPCs can myelinate shiverer organotypic cerebellar slices. (A) Myelin generated by human fetal-derived O4+ oligodendrocyte lineage cells. (B) Myelin generated by human adult-derived O4+ oligodendrocyte lineage cells. Scale bars: 20 µm.

We also assessed whether human oligodendrocyte lineage cells could be utilized in this *in vitro* model of myelination. Transplantation of either human fetal or adult-derived O4-positive oligodendrocyte lineage cells resulted in the successful generation of myelin internodes in the shiverer organotypic cerebellar slices (Figure 6), but this myelination was limited in comparison to that obtained with mouse OPCs. Further optimization of the protocol with these cell types may increase the efficiency of myelination. However, the limited amount of myelin generated may be useful for testing the capacity of compounds to promote myelination by transplanted human oligodendrocyte progenitors.

## Discussion

Our findings demonstrate that OPCs transplanted into organotypic cerebellar slices derived from homozygous shiverer mutant mice generate MBP-positive compact myelin that can be readily distinguished from the MBP-negative membrane ensheathments produced by the shiverer oligodendrocytes. The compact myelin and flanking paranodal domains appear ultrastructurally normal by electron microscopy, although some axons were surrounded by fewer wraps of compact myelin than expected for their given diameter, reminiscent of myelin formed during remyelination [13]. The myelin formed by the transplanted oligodendrocytes resulted in the sequestering of Kv1.2 channels, caspr, and sodium channels into distinct domains at the juxtaparanode, paranode, and node, which were maintained for more than 64 days post-transplant. Taken together, we conclude that this transplant model is applicable to study both myelination and myelin maintenance *in vitro*, providing a time- and cost-effective adjunct to *in vivo* approaches.

A key factor in achieving robust myelination by the transplanted cells was the composition of the organotypic slice culture media. We observed that the commonly used slice culture media containing 25% serum is not optimal for incorporation of transplanted OPCs into the slice. Instead, we utilized a serum-free defined medium, which resulted in a robust increase in the amount of myelination observed. In addition, while the age of the slice at transplant was a flexible variable in this protocol (we transplanted into slices that had been in culture up to 30 days with success), we noted that transplanting into slices that had matured for less than 8 DIV resulted in considerably reduced levels of myelination by the exogenous OPCs. This time point corresponds with when the majority of cell death associated with preparing the slices is complete, suggesting that a stabilized slice environment is beneficial for OPC incorporation into the slice. We also found that mouse OPCs isolated from mixed glial cultures after 8–10 DIV more robustly myelinated the shiverer organotypic slices than mouse OPCs isolated after 12–14 DIV, consistent with younger, less differentiated OPCs, having a greater capacity to myelinate.

Previous studies have reported similar experimental paradigms to myelinate shiverer organotypic slices *in vitro*. Billings-Gagliardi and colleagues [14] showed that organotypic shiverer cerebellar slices injected with explants of wild-type optic nerve exhibited axons surrounded by apparently normal myelin. Additionally, transplantation of rodent and human embryonic stem-cell derived O4-positive oligodendrocyte progenitors into the hippocampus of whole brain organotypic shiverer slices has been used to determine how different pre-treatments of the transplanted cells enhanced myelination [7], [15]. However, in these two studies, the baseline amount of myelination present without treatment was very low in comparison to what we report here, which could be explained by differences both in the source of transplanted OPCs and in the tissue area into which the OPCs were transplanted. Furthermore, while one study did confirm the presence of compact myelin by electron microscopy [15], neither addressed the formation of paranodal junctions or the specialized axonal domains that surround the node of Ranvier.

As a proof of principle we have transplanted wild-type OPCs. However, this model is well-suited to study how mutations or genetic modifications carried specifically by the transplanted oligodendrocytes influence the formation and maintenance of myelin. This will be of utility in scenarios where conventional, but not conditional, knock-out mice are available for study, particularly for mutations that are lethal before myelination is normally well established *in vivo*. While using immunohistochemistry for MBP is an extremely useful tool to identify and study myelin generated by transplanted oligodendrocytes, it is also a potential limitation. MBP is not a practical marker for counting the number of myelinating oligodendrocytes. Traditional markers to label and count oligodendrocyte cells bodies, such as CC1 or olig2, will not distinguish between endogenous shiverer oligodendrocytes and the exogenous transplanted oligodendrocytes. In addition, since MBP expression begins during terminal differentiation of oligodendrocytes [16], this limits observations to after this time point. To examine effects on pre-myelinating oligodendrocytes prior to terminal differentiation, OPCs would need an alternative method to be identified, such as genetic modification to express green fluorescent protein.

In addition to studying genetic mutations, this model could also be used to investigate the effect of pharmacological manipulations on myelination. *In vitro* models of myelination are of particular interest for drug studies targeting autoimmune CNS demyelinating diseases, such as multiple sclerosis, as the absence of the peripheral immune system allows for an assessment of the direct effect of therapies on CNS myelination, which may be confounded using *in vivo* models. Furthermore, the method provides the opportunity to compare and translate findings from rodents to humans using xenotransplantation of oligodendrocyte lineage cells. Transplantation into shiverer slices could be advantageous over testing drugs on regular organotypic slices since transplantation adds the ability to temporally control the onset of the myelination being quantified. In wild-type organotypic slices, endogenous oligodendrocytes express MBP by 3 DIV with myelination following shortly after [3]. During this initial time in culture, the slices undergo a period of cell death and reorganization [4], which could be affected by the addition of a drug and make the effect on myelination more difficult to interpret. This can be avoided by transplanting and treating the slices after they have stabilized in culture. Quantitative measures to screen for agents that promote or inhibit myelination can be readily carried out, for example, by assessing the co-localization of MBP and neurofilament heavy chain using immunohistochemistry [1], [17], complemented by using electron microscopy to verify compact myelin ultrastructure. We anticipate that this model will be beneficial to advance our understanding of the mechanisms that underlie myelin formation and maintenance, with the ultimate goal of promoting remyelination in demyelinating diseases.
